# Investigating genetically mimicked effects of statins via HMGCR inhibition on immune-related diseases in men and women using Mendelian randomization

**DOI:** 10.1038/s41598-021-02981-x

**Published:** 2021-12-03

**Authors:** Guoyi Yang, C. Mary Schooling

**Affiliations:** 1 School of Public Health, Li Ka Shing Faculty of Medicine, The University of Hong Kong, Hong Kong, China; 2 Graduate School of Public Health and Health Policy, City University of New York, New York, USA

**Keywords:** Immunological disorders, Epidemiology, Drug therapy

## Abstract

Statins have been suggested as a potential treatment for immune-related diseases. Conversely, statins might trigger auto-immune conditions. To clarify the role of statins in allergic diseases and auto-immune diseases, we conducted a Mendelian randomization (MR) study. Using established genetic instruments to mimic statins via 3-hydroxy-3-methylglutaryl-coenzyme A reductase (HMGCR) inhibition, we assessed the effects of statins on asthma, eczema, allergic rhinitis, rheumatoid arthritis (RA), psoriasis, type 1 diabetes, systemic lupus erythematosus (SLE), multiple sclerosis (MS), Crohn’s disease and ulcerative colitis in the largest available genome wide association studies (GWAS). Genetically mimicked effects of statins via HMGCR inhibition were not associated with any immune-related diseases in either study after correcting for multiple testing; however, they were positively associated with the risk of asthma in East Asians (odds ratio (OR) 2.05 per standard deviation (SD) decrease in low-density lipoprotein cholesterol (LDL-C), 95% confidence interval (CI) 1.20 to 3.52, *p* value 0.009). These associations did not differ by sex and were robust to sensitivity analysis. These findings suggested that genetically mimicked effects of statins via HMGCR inhibition have little effect on allergic diseases or auto-immune diseases. However, we cannot exclude the possibility that genetically mimicked effects of statins via HMGCR inhibition might increase the risk of asthma in East Asians.

## Introduction

Statins are one of the most commonly used drugs in the world which prevent cardiovascular diseases and reduce mortality^1^. Statins target 3-hydroxy-3-methylglutaryl-coenzyme A reductase (HMGCR) to reduce low-density lipoprotein-cholesterol (LDL-C)^2^. Nevertheless, statins are increasingly recognized as having pleiotropic effects beyond lipid-lowering properties^3,4^. Statins have been suggested as a potential treatment for immune-related diseases, possibly due to their anti-inflammatory and immunomodulatory effects^5,6^. Meta-analysis of randomized controlled trials (RCTs) suggest that statins have anti-inflammatory effects and ameliorate RA activity in RA patients^7,8^. Trial evidence for statins treatment on other immune-related diseases is limited^6,9^.

Conversely, statins are associated with auto-immune myopathies, with the presence of autoantibodies against HMGCR^10,11^, which implies that statins might trigger auto-immune diseases^6^. Observational studies of statins use and risk of developing auto-immune diseases are inconsistent^12–15^. Observational studies of drug side-effects can also be difficult to interpret because of potential nocebo effects^16^ and the possibility of confounding. Trial investigation of whether statins affect risk of auto-immune conditions is very limited. Moreover, few studies are sex-specific although auto-immune conditions tend to be more common in women than men^17^, possibly because of hormone related effects on the immune system^18^. Previous studies have demonstrated that statins affect hormones, possibly more in men than women^19,20^.

To assess the effects of statins via HMGCR inhibition on immune-related diseases, we used a Mendelian randomization (MR) study, i.e., instrumental variable analysis with genetic instruments, which takes advantage of genetic randomization at conception to obtain unconfounded estimates^21^, here sex-specifically. We used established genetic variants to mimic the effects of statins via HMGCR inhibition, applied to the largest available sex-combined genome wide association study (GWAS) and sex-specific genetic summary statistics from the UK Biobank, with Biobank Japan used for replication.

## Results

### Genetic instruments for statins via HMGCR inhibition

Of six SNPs mimicking genetic effects of statins via HMGCR inhibition (rs12916, rs10066707, rs17238484, rs2006760, rs2303152 and rs5909), all SNPs were correlated. In the main analysis, only the lead SNP rs12916, with the strongest association with LDL-C, was used (Supplemental Table S1, *p* value 4.32 × 10^−144^). In sensitivity analysis, all available SNPs and their correlation matrix were included.

The F-statistics for independent SNPs were all > 10 in men, women and overall. None of the SNPs were associated with socioeconomic position, current smoking or alcohol consumed in the UK Biobank. Supplemental Table S1 shows the associations of the SNPs used with LDL-C, as well as the F-statistic for each SNP.

### Associations of genetically mimicked statins via HMGCR inhibition with immune-related diseases

**Figure 1:**
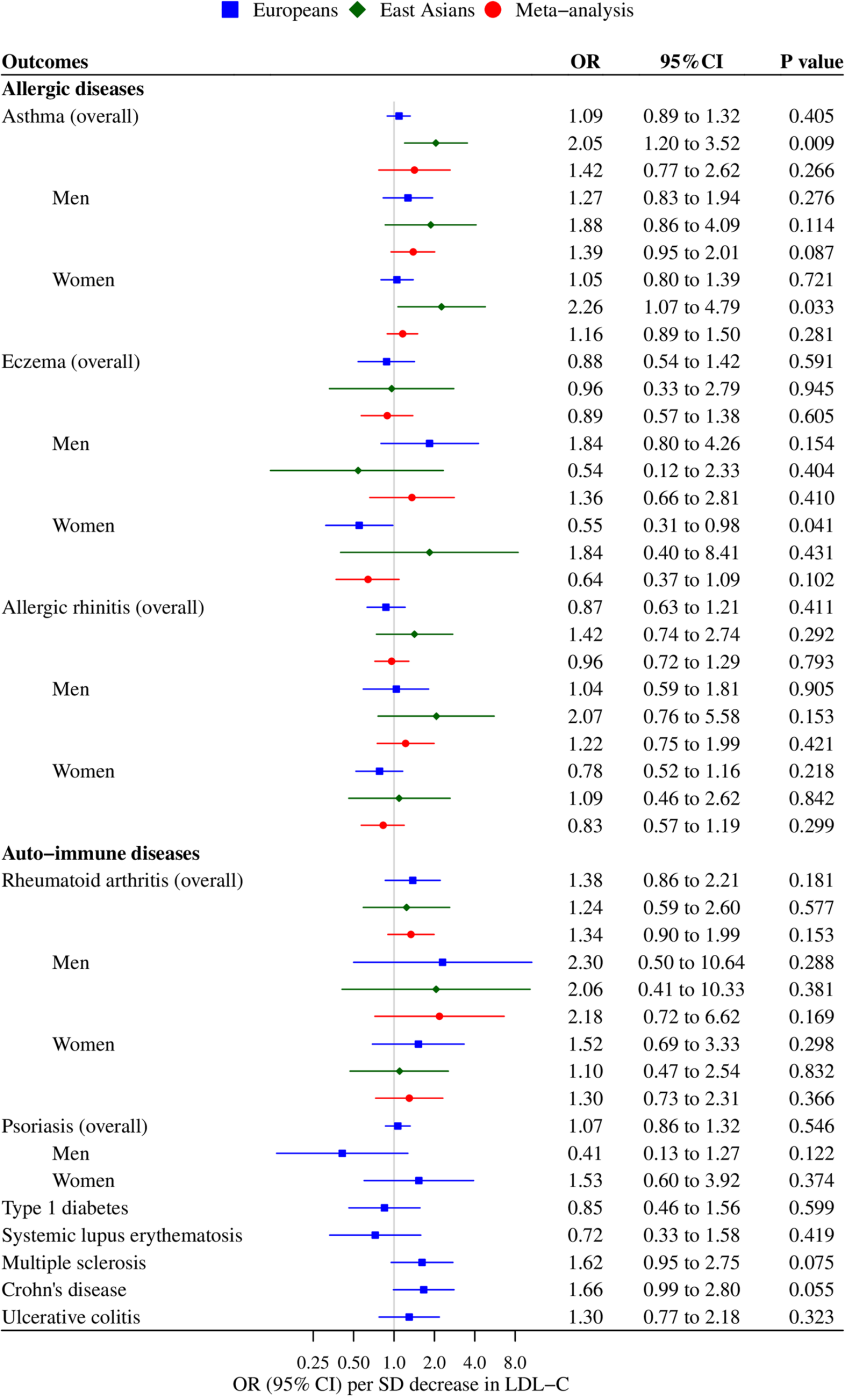
Genetically mimicked effects of statins via HMGCR inhibition (based on rs12916) on allergic and auto-immune diseases. *HMGCR* 3-hydroxy-3-methylglutaryl-coenzyme A reductase, *OR* odds ratio, *CI* confidence interval, *SD* standard deviation, *LDL-C* low-density lipoprotein cholesterol. Only rs12916 was included in the analysis using inverse variance weighted (IVW) method. The unit of LDL-C reduction is approximately 0.87 mmol/L in Europeans and 1.06 mmol/L in East Asians.

Overall and sex-specific associations of genetically mimicked statins via HMGCR inhibition with immune-related diseases are shown in Fig. 1. Genetically mimicked effects of statins via HMGCR inhibition did not affect any allergic diseases or auto-immune diseases in men, women and overall. Genetically mimicked effects of statins via HMGCR inhibition were positively associated with the risk of asthma in East Asians overall (odds ratio (OR) 2.05 per standard deviation (SD) decrease in LDL-C, 95% confidence interval (CI) 1.20 to 3.52, *p* value 0.009), although the *p* value did not reach the Bonferroni corrected significance level. Such an association was not observed in people of European decent (*p* value for ethnic difference 0.030). There was no significant difference in genetically mimicked effects of statins via HMGCR inhibition on immune-related diseases between men and women. These associations were generally robust to sensitivity analysis shown in Supplemental Fig. S1.

This study has 0.8 power to detect an OR of approximately 1.29, 1.70, 1.48, 1.72, 1.95, 2.26, 2.17, 1.87, 1.86 and 1.81 per SD decrease in LDL-C for asthma, eczema, allergic rhinitis, RA, psoriasis, type 1diabetes, SLE, MS, Crohn’s disease and ulcerative colitis in Europeans, respectively. The Biobank Japan has 0.8 power to detect an OR of approximately 1.78, 2.40, 1.92 and 2.07 per SD decrease in LDL-C for asthma, eczema, allergic rhinitis and RA in East Asians, respectively.

## Discussion

This Mendelian randomization (MR) suggests that genetically mimicked effects of statins via HMGCR inhibition have little effect on allergic diseases or auto-immune diseases in men or women. However, we cannot exclude the possibility that genetically mimicked effects of statins via HMGCR inhibition might increase the risk of asthma in East Asians.

Our findings are consistent with a population-based cohort study which investigated overall effects of statins and revealed no change in the risk of RA, psoriasis, SLE or MS^13^. Another prospective cohort study found that use of any type of statins was not associated with the risk of RA in men or women^14^. Our findings are less consistent with case–control studies suggesting a positive or inverse association of statins use with the risk of RA^12,15^ and a retrospective cohort study suggesting that statin use increased the incidence of eczema^22^. However, these studies are subject to bias from unmeasured confounding^12,22^, selection bias^15,22^ and possibly nocebo effects of statins^16^.

Clinical trials of statin treatment in asthmatic patients have yielded contradictory results^23^, probably due to differences in participants’ characteristics (i.e., severity of asthma, age, obesity and smoking status), doses and types of statins. Meta-analysis of RCTs have found that statins suppress inflammation and improve RA symptoms in RA patients^7,8^. An RCT also showed benefits of additional atorvastatin to topical betamethasone in the treatment of chronic hand eczema^24^. However, these trials examined a short-term effect on the prognosis in patients, while we used MR to assess the lifetime effects on the risk of developing immune-related diseases in the general population.

An association of genetically mimicked statins via HMGCR inhibition with asthma was not observed in Europeans, but only in East Asians. The discrepancy might be due to the different study designs of the UK Biobank and Biobank Japan. The UK Biobank had a low response to the baseline survey. However, selection bias occurs in a non-representative study if the study was selected on exposure and outcome. There is no reason to think that both genetic make-up and immune-related conditions determined recruitment into the UK Biobank. Correspondingly, risk factor associations in UK Biobank are similar to those seen in population representative studies, both overall and by sex^25^. Biobank Japan is a multi-institutional hospital-based registry^26^, where the controls are largely patients, rather than being representative of the population that generated the cases. Although the study included not only 179,660 patients but also 32,793 population-based controls^27^, we cannot exclude the possibility that the control selection generated a false positive. Another possibility is that the effects of statins via HMGCR inhibition on asthma might vary by population. Causal effects act consistently, but the mechanism may not be relevant in all settings, thereby resulting in different effects in different settings^28^. Statins largely operate by modulating lipids, but are known to have pleiotropic effects, such as increasing body weight^29^, which is positively associated with the risk of asthma^30^. Japanese are more likely to develop asthma with less weight gain than Europeans^31^, which might explain the discrepancy in the association of genetically mimicked statins via HMGCR inhibition with asthma between East Asians and Europeans.

To our knowledge, our study is the first MR study investigating genetically mimicked effects of statins via HMGCR inhibition on the development of immune-related diseases. Recent GWAS enabled us to examine sex-specific associations in people of European descent and East Asians.

Nevertheless, there are several limitations in the study. First, MR is based on three rigorous assumptions, that is the genetic variants are strongly associated with the exposure, the variants are independent of confounders of the exposure-outcome association and the variants only affect the outcome via effects on the exposure^32^. To satisfy the assumption of relevance, the SNPs used to mimic effects of statins were established on functional ground and were in the relevant target gene (*HMGCR*)^33^. The F-statistics for the SNPs were all > 10, which suggests little weak instrument bias. We checked for the associations of all six SNPs with potential confounders (i.e., socioeconomic position, current smoking and alcohol consumed) in UK Biobank. There was no association of the SNPs with these confounders, which supports the independence assumption. Statins act via HMGCR, but are typically instrumented on LDL-C^33^, as here, because HMGCR is rarely measured and LDL-C is the intended target. However, instrumenting on LDL-C does not imply that LDL-C is the mechanism of action for all the consequences of statins. It just provides an interpretable means of quantifying the MR estimates. As such, examining genetically mimicked effects of statins via HMGCR inhibition on immune-related diseases does not violate the exclusion restriction assumption because we used SNPs in the *HMGCR* gene and then for ease of interpretation provided estimates in terms of changes to LDL-C. Second, although we used the largest available study giving sex-specific genetic associations, the number of cases of some conditions was low, which may explain the relatively wide confidence intervals. We used meta-analysis to combine MR estimates in Europeans and East Asians to increase the power. Third, the genetic instruments for statins via HMGCR inhibition were for both sexes instead of sex-specific instruments in the Biobank Japan. However, there is no reason to think that the genetics of statins via HMGCR inhibition differ by sex, although the consequences might do. Fourth, MR can be open to selection bias due to only selecting survivors. We cannot exclude the possibility that some participants eligible for UK Biobank could not be recruited because they had already died^34^. However, the immune-related conditions considered are rarely fatal, and thus these estimates are less likely to be biased. Fifth, population stratification might affect the MR estimates. However, the underlying studies were corrected for population stratification. Sixth, canalization might occur, which means that genetic effects might be buffered during development. Nevertheless, it would not explain the positive association of genetically mimicked statins via HMGCR inhibition with asthma in East Asians, since such complementary mechanisms generally mitigate genetic effects and bias towards the null. Seventh, Rho/Rho-associated coiled-coil-containing protein kinase (ROCK), Rac, Ras and peroxisome proliferator-activated receptors (PPARs) could also account for pleiotropic effects of statins^4,35^. However, these proteins are downstream of the HMGCR pathway. We cannot exclude the possibility that statins act via other pathways unrelated to HMGCR inhibition, which we could not investigate here because such pathways remain to be identified.

In conclusion, genetically mimicked effects of statins via HMGCR inhibition have little effect on allergic diseases or auto-immune diseases. However, we cannot exclude the possibility that genetically mimicked effects of statins via HMGCR inhibition might increase the risk of asthma in East Asians.

## Methods

### Genetic predictors mimicking effects of statins via HMGCR inhibition

Established genetic predictors of the effects of statins via HMGCR inhibition were used, i.e., six single nucleotide polymorphisms (SNPs) from the *HMGCR* gene^33^. Independent genetic mimics of effects of statin via HMGCR inhibition (r^2^ < 0.01) most strongly associated with LDL-C were used in the main analysis, while all available SNPs along with their correlation matrix were included in sensitivity analysis. Sex-combined and sex-specific effects of genetically mimicked statins via HMGCR inhibition were reported in terms of a standard deviation (SD) decrease in LDL-C taken from the largest sex-specific genetic summary statistics, i.e., UK Biobank (http://www.nealelab.is/uk-biobank/) and Biobank Japan^36^. The UK Biobank study included 361,194 people of white British ancestry aged 40–69 years (194,174 women and 167,020 men). Associations from linear regression were adjusted for age, age^2^, inferred sex, age × inferred sex, age^2^ × inferred sex and the first 20 principal components. The Biobank Japan study included 72,866 Japanese individuals (42.8% women) with a mean age of 63.9 years, adjusted for age, sex, the top 10 principal components and status of 47 diseases.

To assess potential pleiotropy, associations of all six SNPs with potential confounders (i.e., Townsend index for socioeconomic position, current smoking and alcohol consumed) were also checked in the UK Biobank.

### Genetic associations with immune-related diseases in Europeans

Outcomes included allergic diseases (i.e., asthma, eczema and allergic rhinitis) and auto-immune diseases (i.e., RA, psoriasis, type 1 diabetes, systematic lupus erythematosus (SLE), multiple sclerosis (MS), Crohn’s disease and ulcerative colitis). We obtained sex-combined genetic associations with each immune-related disease from the largest publicly available GWAS^37–43^. Information about relevant GWAS is summarized in Supplemental Table S2.

Sex-specific genetic associations with asthma, eczema, allergic rhinitis, RA and psoriasis in people of European descent were obtained from UK Biobank summary statistics (http://www.nealelab.is/uk-biobank/). Cases were defined based on self-reported illness. Genetic associations for all or nothing outcomes obtained using linear regression were transformed into log odds ratio (OR) using an established approximation^44^.

### Genetic associations with immune-related diseases in East Asians

Both sex-combined and sex-specific genetic associations with immune-related diseases in East Asians were obtained from a GWAS of Biobank Japan for replication, where applicable^27^. The GWAS included 179,660 patients in Biobank Japan and 32,793 population-based controls. The Biobank Japan study is a multi-institutional hospital-based registry, which recruited patients with newly developed diseases and also patients who were diagnosed and treated before the study started^26^. Age, sex and the top five principal components were adjusted for in the analysis, using scalable and accurate implementation of generalized mixed model (SAIGE).

### Statistical analysis

The F-statistic was used to assess the strength of the genetic instruments, approximated by the mean of the square of each SNP-exposure association divided by the square of its standard error^45^. An F-statistic larger than 10 suggests weak instrument bias is unlikely.

MR estimates were obtained by meta-analyzing Wald estimates (ratio of SNP on outcome to SNP on exposure) using inverse variance weighting (IVW) with fixed effects for three SNPs or less or random effects for four SNPs or more. In sensitivity analysis, all relevant SNPs were used with a matrix of their correlations, obtained by using the “ld_matrix” function from MRBase. MR estimates in Europeans and East Asians were subsequently meta-analyzed using a fixed-effects model unless the Q-statistic suggested heterogeneity when random effects were used. To examine potential pleiotropy we assessed whether the genetic instruments were associated with key potential confounders at genome wide significance in the UK Biobank.

Power calculations were conducted based on the approximation that the sample size required for an MR study is the sample size for exposure on outcome divided by the R^2^ for instrument on exposure^46^. The R^2^ for instrument on exposure was estimated as 2 × beta^2^ × MAF × (1 − MAF), where beta is the genetic association with the exposure in SD units and MAF is the minor allele frequency.

A Bonferroni corrected significance level was set at α = 0.05/10 = 0.005, where 10 was the number of phenotypes included. All statistical analyses were conducted using R version 4.0.3 and the packages “MendelianRandomization”, “TwoSampleMR” and “metafor”. Results were visualized using the package “forestplot”. All analyses were based on publicly available data, which does not require ethical approval.

## Supplementary Information

Supplementary Information.

## Data Availability

The datasets analyzed during the current study are available in UK Biobank website (http://www.nealelab.is/uk-biobank/), Biobank Japan website (http://jenger.riken.jp/en/result/), and GWAS catalogue website (https://www.ebi.ac.uk/gwas/).
