# Supplementary material for: Investigating genetically mimicked effects of statins via HMGCR inhibition on immune-related diseases in men and women using Mendelian randomization
Source: Sci Rep. 2021 Dec 3;11:23416. doi: 10.1038/s41598-021-02981-x (PMC8642420; doi:10.1038/s41598-021-02981-x)
Supplement: Supplementary file 1 — Supplementary Information. [file 41598_2021_2981_MOESM1_ESM.pdf]

Europeans ◆ East Asians ● Meta−analysis

Outcomes	SNP	OR	95%CI	P value
**Allergic diseases**				
Asthma (overall)	6	1.05	0.83 to 1.33	0.661
	5	2.11	1.30 to 3.43	0.003
	/	1.44	0.73 to 2.83	0.291
Men	6	1.18	0.61 to 2.29	0.629
	5	2.04	1.01 to 4.13	0.047
	/	1.53	0.94 to 2.47	0.087
Women	6	1.02	0.70 to 1.47	0.931
	5	2.21	1.12 to 4.35	0.022
	/	1.42	0.67 to 3.01	0.363
Eczema (overall)	6	0.92	0.58 to 1.46	0.719
	5	1.07	0.41 to 2.81	0.892
	/	0.94	0.62 to 1.43	0.790
Men	6	1.76	0.74 to 4.23	0.203
	5	0.96	0.12 to 7.47	0.966
	/	1.61	0.72 to 3.59	0.249
Women	6	0.62	0.36 to 1.07	0.088
	5	1.21	0.19 to 7.73	0.838
	/	0.65	0.39 to 1.11	0.114
Allergic rhinitis (overall)	6	0.84	0.61 to 1.15	0.283
	5	1.01	0.42 to 2.42	0.990
	/	0.86	0.64 to 1.16	0.314
Men	6	1.04	0.61 to 1.78	0.889
	5	1.32	0.40 to 4.34	0.643
	/	1.08	0.66 to 1.77	0.751
Women	6	0.74	0.50 to 1.08	0.119
	5	0.83	0.35 to 1.95	0.672
	/	0.75	0.53 to 1.07	0.111
**Auto−immune diseases**				
Rheumatoid arthritis (overall)	6	1.41	0.88 to 2.26	0.155
	5	1.13	0.57 to 2.22	0.728
	/	1.31	0.89 to 1.93	0.173
Men	6	1.72	0.39 to 7.48	0.472
	5	2.29	0.53 to 9.92	0.267
	/	1.98	0.70 to 5.61	0.196
Women	6	1.44	0.68 to 3.06	0.340
	5	0.94	0.44 to 2.02	0.879
	/	1.17	0.68 to 2.00	0.568
Psoriasis (overall)	2	1.07	0.86 to 1.32	0.546
Men	6	0.38	0.11 to 1.27	0.116
Women	6	1.73	0.70 to 4.27	0.235
Type 1 diabetes	2	0.83	0.45 to 1.52	0.545
Systemic lupus erythematosis	3	0.74	0.34 to 1.60	0.442
Multiple sclerosis	2	1.60	0.94 to 2.71	0.084
Crohn's disease	5	1.58	0.96 to 2.60	0.072
Ulcerative colitis	5	1.41	0.70 to 2.84	0.340

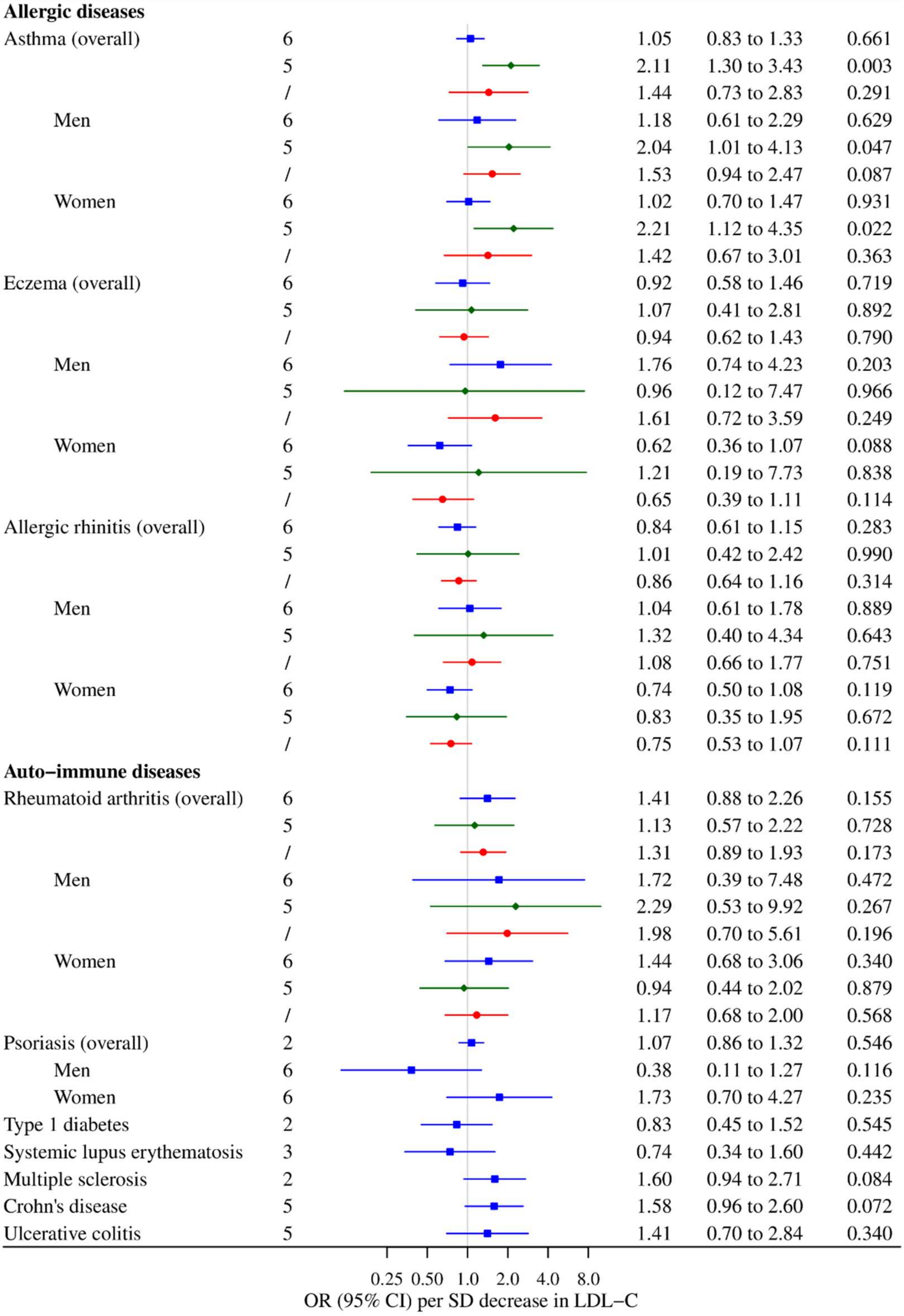

Supplemental Fig. S1. Sensitivity analysis of genetically mimicked effects of statins via HMGCR inhibition on allergic and auto-immune diseases.

HMGCR, 3-hydroxy-3-methylglutaryl–coenzyme A reductase; OR, odds ratio; CI, confidence interval; SD, standard deviation; LDL-C, low-density lipoprotein cholesterol. All correlated SNPs and their correlations were included in the analysis using inverse variance weighted (IVW) method. The unit of LDL-C reduction is approximately 0.87 mmol/L in Europeans and 1.06 mmol/L in East Asians.

Supplemental Table S1. Associations of SNPs mimicking statins via HMGCR inhibition with LDL-C from the UK Biobank and Biobank Japan.

Population	Source	Sex	SNP	Effect allele	Beta	SE	P-value	F-statistics
European	UK Biobank	Overall	rs12916	C	0.0614	0.0024	4.32E-144	653.9
			rs10066707	A	0.0404	0.0024	3.33E-61	272.5
			rs17238484	T	0.0508	0.0028	7.42E-73	326.1
			rs2006760	G	0.0347	0.0030	1.12E-31	137.2
			rs2303152	A	0.0316	0.0039	8.54E-16	64.7
			rs5909	A	0.0480	0.0040	1.99E-32	140.6
	UK Biobank	Men	rs12916	C	0.0527	0.0036	2.45E-49	218.2
			rs10066707	A	0.0363	0.0036	1.68E-23	99.8
			rs17238484	T	0.0444	0.0042	2.44E-26	112.8
			rs2006760	G	0.0302	0.0044	7.08E-12	47.0
			rs2303152	A	0.0294	0.0058	4.33E-07	25.5
			rs5909	A	0.0391	0.0060	8.37E-11	42.2
	UK Biobank	Women	rs12916	C	0.0695	0.0033	3.25E-100	452.2
			rs10066707	A	0.0442	0.0033	4.19E-40	175.8
			rs17238484	T	0.0568	0.0038	6.97E-50	220.6
			rs2006760	G	0.0390	0.0040	3.51E-22	93.8
			rs2303152	A	0.0342	0.0053	1.67E-10	40.8
			rs5909	A	0.0561	0.0055	2.68E-24	103.5
East Asian	Biobank Japan	Overall	rs12916	C	0.0584	0.0052	1.91E-29	127.0
			rs10066707	A	0.0293	0.0054	4.85E-08	29.8
			rs17238484	T	0.0553	0.0055	4.82E-24	102.3
			rs2303152	A	0.0403	0.0100	5.20E-05	16.4
			rs5909	A	0.0811	0.0224	2.96E-04	13.1

HMGCR, 3-hydroxy-3-methylglutaryl–coenzyme A reductase; LDL-C, low-density lipoprotein cholesterol.

Supplemental Table S2. Information about genome-wide association studies involved.

Population	Outcomes	Sex	No. of cases	No. of controls	Beta transformation	Data source
European	Asthma	Overall	64538	329321	No	Han Y (PMID: 32296059)
		Men	17973	149015	Yes	UK Biobank (Neale Lab)
		Women	23961	170192	Yes	UK Biobank (Neale Lab)
	Eczema	Overall	9321	351820	Yes	UK Biobank (Neale Lab)
		Men	4255	162733	Yes	UK Biobank (Neale Lab)
		Women	5066	189087	Yes	UK Biobank (Neale Lab)
	Allergic rhinitis	Overall	20667	340474	Yes	UK Biobank (Neale Lab)
		Men	9704	157284	Yes	UK Biobank (Neale Lab)
		Women	10963	183190	Yes	UK Biobank (Neale Lab)
	Rheumatoid arthritis	Overall	14361	43923	No	Okada Y (PMID: 24390342)
		Men	1259	165729	Yes	UK Biobank (Neale Lab)
		Women	2758	191395	Yes	UK Biobank (Neale Lab)
	Psoriasis	Overall	10588	22806	No	Lam C Tsoi (PMID: 23143594)
		Men	2268	164720	Yes	UK Biobank (Neale Lab)
		Women	1924	192229	Yes	UK Biobank (Neale Lab)
	Type 1 diabetes	Overall	12079	12262	No	Onengut-Gumuscu S (PMID: 25751624)
	Systemic lupus erythematosis	Overall	7219	15991	No	Bentham J (PMID: 26502338)
	Multiple sclerosis	Overall	14498	24091	No	Beecham AH (PMID: 24076602)
	Crohn's disease	Overall	12194	28072	No	de Lange KM (PMID: 28067908)
	Ulcerative colitis	Overall	12366	33609	No	de Lange KM (PMID: 28067908)
East Asian	Asthma	Overall	8216	201592	No	Biobank Japan
		Men	3932	103089	No	Biobank Japan
		Women	4284	98503	No	Biobank Japan
	Eczema	Overall	2385	209651	No	Biobank Japan
		Men	1262	107862	No	Biobank Japan
		Women	1123	101789	No	Biobank Japan
	Allergic rhinitis	Overall	5746	206707	No	Biobank Japan
		Men	2460	106887	No	Biobank Japan
		Women	3286	99820	No	Biobank Japan
	Rheumatoid arthritis	Overall	4199	208254	No	Biobank Japan
		Men	874	108473	No	Biobank Japan
		Women	3325	99781	No	Biobank Japan
